# An Automated Toolchain for Camera-Enabled Sensing of Drinking Water Chlorine Residual

**DOI:** 10.1021/acsestengg.2c00073

**Published:** 2022-06-03

**Authors:** Alyssa Schubert, Leah Pifer, Jianzhong Cheng, Shawn P. McElmurry, Branko Kerkez, Nancy G. Love

**Affiliations:** † Department of Civil and Environmental Engineering, University of Michigan, Ann Arbor, Michigan 48109, United States; ‡ Department of Civil and Environmental Engineering, Wayne State University, Detroit, Michigan 48202, United States

**Keywords:** free chlorine, monitoring, automated processing

## Abstract

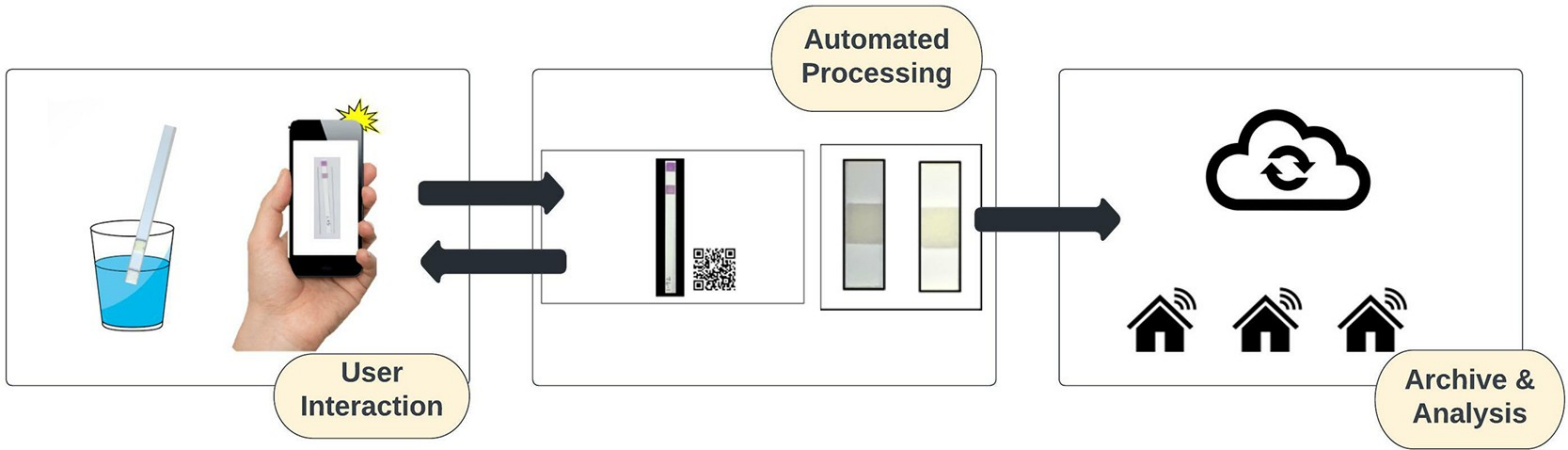

Chlorine residual concentration is an important parameter to prevent pathogen growth in drinking water. Disposable color changing test strips that measure chlorine in tap water are commercially available to the public; however, the color changes are difficult to read by eye, and the data are not captured for water service providers. Here we present an automated toolchain designed to process digital images of free chlorine residual test strips taken with mobile phone cameras. The toolchain crops the image using image processing algorithms that isolate the areas relevant for analysis and automatically white balances the image to allow for use with different phones and lighting conditions. The average red, green, and blue (RGB) color values of the image are used to predict a free chlorine concentration that is classified into three concentration tiers (<0.2 mg/L, 0.2–0.5 mg/L, or >0.5 mg/L), which can be reported to water users and recorded for utility use. The proposed approach was applied to three different phone types under three different lighting conditions using a standard background. This approach can discriminate between concentrations above and below 0.5 mg/L with an accuracy of 90% and 94% for training and testing data sets, respectively. Furthermore, it can discriminate between concentrations of <0.2 mg/L, 0.2–0.5 mg/L, or >0.5 mg/L with weighted-averaged F1 scores of 79% and 88% for training and testing data sets, respectively. This tool sets the stage for tap water consumers and water utilities to gather frequent measurements and high-resolution temporal and spatial data on drinking water quality.

## 1 Introduction

Free chlorine is a disinfectant commonly used in the treatment of drinking water to protect public health. It is widely applied to inactivate pathogens that may be present in source water^1^ and to prevent microbial regrowth and recontamination as finished drinking water travels through the water distribution system (WDS). The WDS is a network of pipes and storage structures that conveys treated drinking water from the outlet of the treatment plant and to the service line via water mains and ultimately to premise plumbing, which carries water from the service line to the tap (point-of-use) within buildings and residences. While drinking water utilities do not have direct authority in premise plumbing, water quality in premise plumbing, including chlorine residual concentration, is a function of water quality in the water mains. Monitoring residual chlorine concentrations in the total WDS (mains and premise plumbing) is critical to understanding water quality. However, current compliance monitoring practices regulate chlorine residual concentrations at a limited number of nodes throughout the WDS. Furthermore, few water utilities track water quality in premise plumbing, despite the fact that exposure occurs at the tap. There is growing evidence indicating that water quality at the point-of-use changes substantially as a function of premise plumbing conditions.^1−6^

Maintenance of adequate chlorine residual in drinking water is influenced by a complex list of factors. While the desired residual chlorine concentration varies spatially and temporally from water system to water system for a variety of reasons, including source water characteristics, treatment contact time, and size of the water system,^7^ most states in the U.S. mandate a minimum free chlorine residual in the WDS of either 0.2 or 0.5 mg/L as Cl_2_.^8,9^ In all states, free chlorine residual must remain below the maximum residual disinfectant level (MRDL) of 4.0 mg/L to minimize formation of disinfectant byproducts (DBPs) and taste or odor complaints.^10^ The tolerance of waterborne pathogens to free chlorine varies,^11^ and the opportunistic pathogens that are most prevalent in premise plumbing are not the same as those found in the water mains,^12^ further motivating the necessity for ensuring adequate free chlorine in premise plumbing. For example, prior work examining the relationship between WDS free chlorine residual and incidents of Legionnaires’ Disease caused by the bacterium *Legionella pneumophila* suggests that a residual of 0.2–0.5 mg/L Cl_2_ is insufficient for public health.^13−15^

The Stage 1 Disinfectants and Disinfection Byproduct Rule (DBPR) requires that compliance monitoring samples be taken at the same time and locations where total coliforms are measured.^10^ The Revised Total Coliform Rule requires sampling sites to be accessible and representative of water quality conditions across the water system and, therefore, may include sampling in residential or commercial premise plumbing.^16,17^ However, not all locations in a water system can be monitored, and residential premise plumbing may not be easily accessible, resulting in a data set that is likely too limited in time and space to characterize all portions of a water system.^18^ The success of chlorine residual to prevent the survival or regrowth of most pathogens is heavily influenced by water main pipe age, condition, and material. Importantly, virtually all of the mechanisms by which chlorine decay occurs in water mains are present and possibly magnified in premise plumbing due to the high surface area to volume ratio, potential for high water age, potential mix of pipe materials, and variable velocities and temperatures; these factors can cause the rapid loss of free chlorine in premise plumbing.^3^ Therefore, the public health risk related to low chlorine residual may not be adequately captured by sampling—even frequent sampling—in water mains. Sparse data sets of chlorine residual concentrations in premise plumbing may result in underestimating the risk to water users or overestimating public health safety due to the lack of clarity around water quality throughout all points in the system, especially points beyond the service connection.

Increasingly, water crises are occurring in aging and under-maintained centralized, distributed water systems both domestically and internationally^19−21^ and emphasize the need for water users to have access to and agency in local decision-making. This community–utility communication and engagement process should be based on frequent and accurate information about the quality of the water that consumers purchase and use. Both users and utilities will benefit from having ways to document water quality and to understand how water quality varies by location and time across a system.^22,23^

Studies have shown that lack of user confidence in drinking water quality can contribute to anxiety, stress, and increased spending on alternative sources of drinking water, particularly for water users living in shrinking population cities or underinvested areas.^24−29^ A simple means to test water quality at home is measuring chlorine residual concentration. Free and total chlorine test strips that change color based on chlorine concentration are accessible and commercially available; while regarded as easy to use, they have low user confidence due to the subjectivity involved in determining chlorine concentration by eye,^30,31^ and the current data captured by water users at home are not collected for other use or analysis. Prior work suggests that improved quantity and resolution of water quality data benefits decision makers^6,32,33^ and water users^34^ and provides the opportunity to capture spatial or temporal trends that would be lost with more periodic sampling.^2^ We hypothesize that improving the temporal and spatial variability in chlorine residual at relevant concentrations across a water system will lead to an improved assessment of the public health risk.

Mobile phone camera-based colorimetric analysis has been established as a promising and easily accessible method of sensing for other analytes.^35−40^ A prior study demonstrated the feasibility of mobile phone-based colorimetric field analysis for measuring free chlorine using an illumination sensor coupled to a mobile phone rather than using a built-in camera to measure absorbance.^41^ Select brands of commercially available chlorine residual test strips offer mobile phone camera-based analysis of the test strip using an application (e.g., Clorox) but do not offer analysis at concentrations below 0.5 mg/L as Cl_2_ or provide a means to rapidly integrate data over time and space to produce information that addresses questions about water quality of importance to both community users and utility personnel. Here, we apply a mobile phone camera-based colorimetric analysis approach with two novel objectives: (1) to use camera-based analysis to reliably predict when chlorine residual drops below 0.5 mg/L as Cl_2_, which is more sensitive than is currently achieved with image-based test strip analysis applied to swimming pools, and (2) to automate the measurement process in a way that reduces error and enhances the ability to share and utilize crowdsourced data. To achieve this, we propose a method for digital photobased colorimetric analysis of free chlorine residual using test strips and the cameras of different types of phones against a standard background. First, we developed an automated toolchain to process data obtained from the test strips. Then, we evaluated the toolchain’s performance to predict free chlorine at minimum concentrations typically applied by utilities using polynomial regression and two binning structures, binary and multiclass. Lastly, we used conditional probability analysis to evaluate the toolchain’s ability to reliably provide field measurements of chlorine at concentrations lower than previously reported using other mobile test strip techniques.

## 2 Materials and Methods

**Figure 1:**
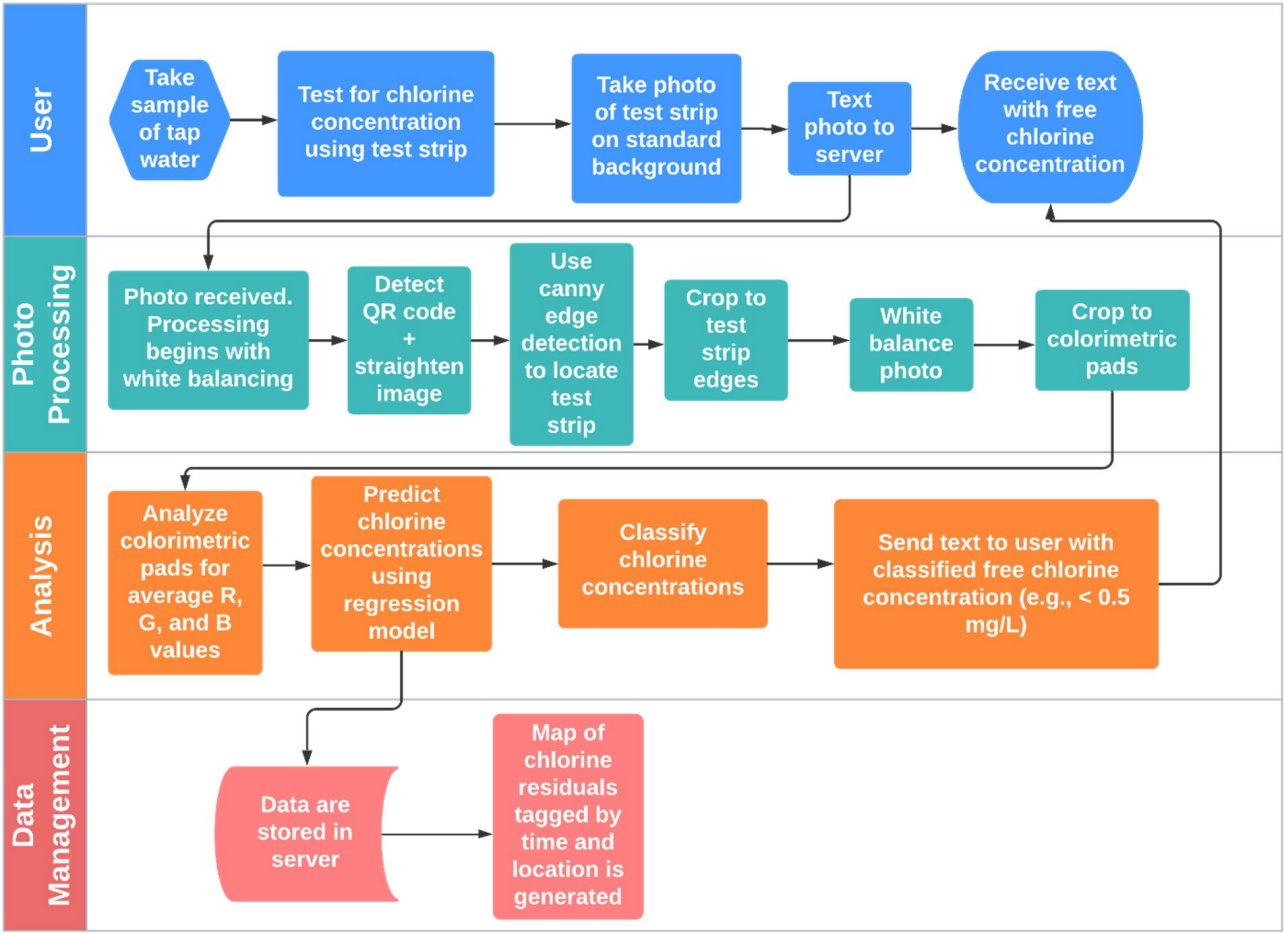
Toolchain flow diagram. The toolchain starts with the user, flows to photo processing and analysis, and the resulting data diverges both to the user and to data management for storage.

The following sections provide further detail about the methods used to develop and test the toolchain in the same order as is presented in Figure 1. First, water samples were prepared in the lab, test strips were dipped in samples, and photos were taken of each test strip; these captured photos were stored in a shared server and split into training data (with which the model was trained) and testing data (to validate model performance). All images were processed and colorimetrically analyzed. Then, we developed our model using the training data. We assessed the performance of the toolchain based on the ability to automate image processing, predict and classify concentrations, and withstand variable conditions. These performance metrics were validated using our testing data. The development process was iterative and is described in detail in the Results and Discussion section.

### 2.1 Sample Preparation and Data Capture

Standard solutions of 2.0 mg/L Cl_2_ were prepared using 50–75 mg/L Cl_2_ free chlorine ampules (Hach Product #1426810, Loveland, CO) diluted with Milli-Q water (>18 mΩ/cm) in acid-washed glassware. The 2.0 mg/L Cl_2_ standard solutions were diluted four times to produce 50 mL solutions with concentrations of 1.0, 0.5, 0.25, and 0.125 mg/L Cl_2_, respectively. Total and free chlorine concentrations of each solution were validated and recorded using a DR 900 Hach colorimeter (Hach, Loveland, CO) and DPD (*N*,*N*-diethyl-*p*-phenylenediamine, a compound that is oxidized in the presence of chlorine) powder pillows (USEPA DPD method 8021 for free chlorine and 8167 for total chlorine, Hach Product #2105528 and #2105669). Once validated, the free and total chlorine concentration of each standard solution was measured using chlorine residual test strips (Hach Product #2745050). Although not an official USEPA method, the chlorine residual test strips used in this work are imbued with DPD (free chlorine colorimetric pad) and DPD with potassium iodide (total chlorine colorimetric pad), which produced a magenta color whose intensity is directly proportional to the chlorine concentration, as described in Standard Method 4500-Cl G.^42^ The test strips were submerged according to the manufacturer’s instructions, removed from solution, and placed on a standard background. The test strips used in this study provided a standard color comparator for concentrations of 0 mg/L, 0.5 mg/L, 1.0 mg/L, 2.0 mg/L, 4.0 mg/L, and 10 mg/L.

It should be noted that while data were collected for both free and total chlorine concentrations, standard solutions were made only using free chlorine (not combined chlorine) ampules. Therefore, we only elucidate free chlorine observations in the Results and Discussion section. Total chlorine results can be found in SI Section S4.

**Figure 2:**
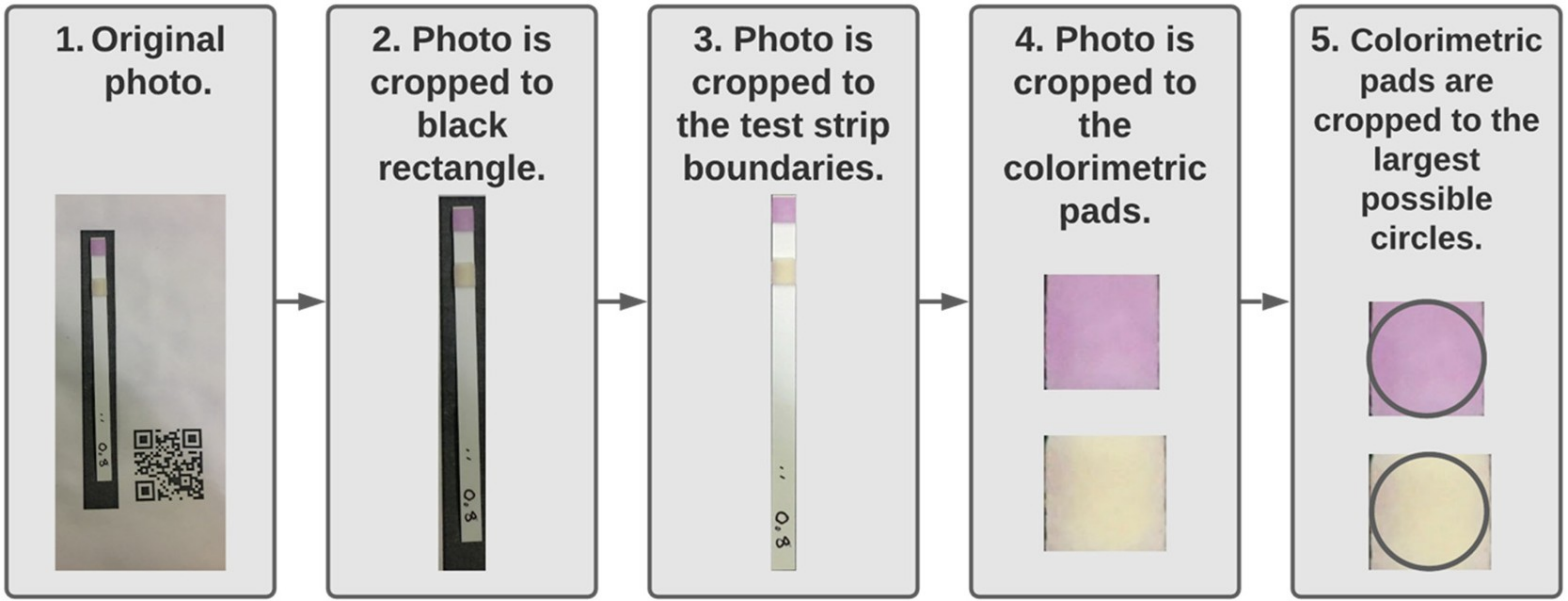
Photo processing flow diagram. First, we used Canny edge detection to locate the black rectangle. Second, masking was used to crop to the test strip edges. Third, the colorimetric pads were cropped. Fourth, the colorimetric pads were cropped to the largest possible circles. White balancing occurs after boxes 1 and 3. The total chlorine pad is at the top end of the strip in these images; the free chlorine pad is below and toward the middle of each test strip.

The standard background, a concept commonly used with image processing,^36^ consisted of a black rectangle approximately three times the width of the test strip, to allow for some variation in test strip placement, and a QR code (Figure 2, box 1). In all data collection efforts, attempts were made to take photos normal to the test strip plane and out of shadow. To test toolchain reliability under variable conditions, photos were taken with an iPhone 5 (rear camera resolution of 8 megapixels or MP), iPhone 8 (rear camera resolution of 12 MP), and LG enV2 (rear camera resolution of 2 MP) and in three lighting conditions (laboratory, hallway, and outdoor). Phones were selected to represent the range of high, average, and low camera resolutions that may be used with the toolchain. Each photo was taken within 2 min of the test strip being submerged to limit variability in the color intensity present on the colorimetric pad.

### 2.2 Training the Toolchain

To train the toolchain and ensure all possible concentrations were included in the training data set, 67% (*n* = 129) of the photos in the complete data set (*n* = 192) were selected to train the toolchain. The photos in the training data set were selected while preserving the relative proportionality of photos within Groups 1–6 in the full data set yet were randomly selected within those groups (Table 1). The remaining 33% of photos, which were not included in the training data set, served as the testing data set (*n* = 63). Model results were cross validated by random resampling to generate 10 training and testing data sets (see SI section S3).

**Table 1:** Number of Photos in the Total and Training Data Sets^a^

group	known concentration (mg/L)	complete data set	training data set
1	0.0 < *C* ≤ 0.125	21	14
2	0.125 < *C* ≤ 0.2	27	18
3	0.2 < *C* ≤ 0.5	54	36
4	0.5 < *C* ≤ 1.0	36	24
5	1.0 < *C* ≤ 2.0	46	31
6	*C* > 2.0	8	6
total	all concentrations	192	129

a Relative proportionality of each group was preserved.

### 2.3 Colorimetric Analysis and Photo Processing

Digital images were colorimetrically analyzed by extracting the color data present in the image. In the RGB (red, green, and blue) additive model, each color data point, or pixel, is made up of three channels (red, green, and blue) combined to create an image; therefore, each pixel is associated with a triplet of numbers (*R*, *G*, *B*) where each number has a distinct value between 0 and 255, referred to as a color value. A larger value indicates more of that color is present. The average red, green, and blue color values for an entire image can be extracted and used in a predictive model as the explanatory variables; the process by which RGB color values were extracted here includes the following steps described in detail.

All photos were processed using the automated toolchain written in Python, primarily using the OpenCV package.^43^ A well-known method to correct for variation in lighting and to prepare the digital photo for further steps is white balancing, which may be approached in a variety of ways.^35^ Here, the entire photo was white balanced using the gray world assumption, which assumes that the average of all colors in an image is gray^44,45^ (eq 1). Each pixel was normalized as shown in eq 2.
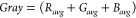
1
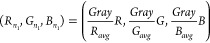
2where *R_avg_*, *G_avg_*, and *B_avg_*, are the average red, green, and blue color values of all pixels in the image, respectively, and *Gray* is the sum of these values; *R*, *G*, and *B* are the red, green, and blue color values in a given pixel; *R*_*n*_1__, *G*_*n*_1__, and *B*_*n*_1__, are the normalized *R*, *G*, and and *B* color values in the given pixel. These values are stored as unitless integers.

Second, the QR code was located to correct the alignment of the photo and give an approximate location of the black rectangle on the standard background (Figure 2). The QR code extraction process consists of applying a Gaussian blur^46^ (a low-pass filter used to remove noise in an image) and Canny edge detection^47^ (a well-known multistage algorithm that can detect the edges in an image), to find the necessary contours of the QR code corners. The corner vertices are used to align the photo. A full discussion of how this QR code extraction process works can be found at the cited GitHub repository.^48^

Third, the black rectangle on which the test strip was placed was located using Canny edge detection to first locate the rectangle contours and then the vertices.^47^ Once the black rectangle was located, the photo was cropped to the test strip edges using masking, in which only the region of interest in an image is extracted.^46^ Then, the test strip was white balanced again by normalizing the average RGB color values in the image relative to the ratio between the theoretical maximum white color values (255, 255, 255) and average white RGB color values (eq 3).
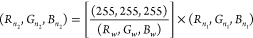
3where *R_w_*, *G_w_*, and *B_w_* are the average *R*, *G*, and *B* color values for the white pixels in the image; *R*_*n*_1__, *G*_*n*_1__, and *B*_*n*_1__ are the *R*, *G*, and *B* normalized gray world assumption color values for a given pixel; *R*_*n*_2__, *G*_*n*_2__, and *B*_*n*_2__ are the *R*, *G*, and *B* color values for a given pixel derived by dividing the average white pixel color values by 255 and multiplying this result by *R*_*n*_1__, *G*_*n*_1__, and *B*_*n*_1__.

Using the masking method, each colorimetric pad was isolated and cropped to the largest possible circle within the pad, which was drawn based on the pad’s center point. The average red, green, and blue color values of the pad were extracted from within the circle bounds. The extracted red, green, and blue color values were explanatory variables in the selected model. The complete code used to process each image is open source and can be found on Github.^49^

### 2.4 Model and Classification Evaluation Metrics

Various statistical measures were employed to evaluate model performance. The regression model fit was evaluated using the *R*-squared statistic and fitted vs residuals plots. Classification performance was evaluated using accuracy (the fraction of total samples correctly predicted by the model), precision (the fraction of true positives out of all predicted positive samples), recall (also known as sensitivity, the fraction of true positives that were correctly predicted positive by the model), and F1-score (a combination of precision and recall into a single metric). The F1-score is a measure of accuracy used to evaluate model performance when the number of samples in each class is unbalanced.^50^ Classification performance was evaluated using both binary and multiclass binning structures. Therefore, accuracy was the primary evaluation metric for the binary binning, and a weighted-averaged F1-score, in which the F1 score for each class is weighted by the number of samples of each class, was calculated as the primary evaluation metric for the multiclass binning.

The performance of the binnings was evaluated using the following statistical metrics: TP (true positive), FP (false positive), TN (true negative), FN (false negative), Ac (accuracy), Pr (precision), Re (recall), F1-score, and Micro F1-score.
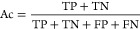
4
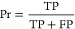
5
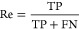
6
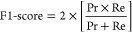
7
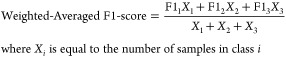
8

## 3 Results and Discussion

### 3.1 At Least 75% of Photos Were Automatically Processed, with All Errors Related to Locating the QR Code

The photos in the training data set were directed to pass through the automated image processing chain via the Python script. A successfully processed photo generated a white balanced, cropped image centered on the colorimetric pad from which the average red, green, and blue color values were extracted. If a photo was not successfully processed, the red, green, and blue color values were not obtained. Of the 129 photos in the training data set, 32 photos were not processed (25%). All processing failures were attributed to failure to locate the QR code, a step that occurs prior to extracting the average RGB values from a photo. Importantly, 25 of the 32 failed photos were taken with the LG enV2 mobile phone. Upon visual inspection of the failed photos, it is hypothesized that the age of the phone is likely the limiting factor. The LG enV2 photos were more pixelated than the photos taken by either iPhone; therefore, it is suspected that the resolution of the photo was not high enough for the QR code to be located correctly.

While it is estimated that over 5 billion people own a mobile phone globally,^51^ this finding has implications for toolchain deployment in areas where mobile phones are older or where mobile devices, but not smartphones, are more ubiquitous. Research suggests that residents in more advanced economies are more likely to own either a mobile phone or smartphone than those in emerging economies;^52^ education and literacy level, gender, and age have also been found to influence mobile phone ownership.^53^ To address and prevent further contribution to this digital divide, modifications may need to be made to improve toolchain performance, such as using a different type of two-dimensional matrix code or improving perspective distortion.^54^ Alternatively, photos could be received to the server and manually cropped prior to entering the toolchain. In this case, we elected to continue training with 97 photos that were completely processed with the automated toolchain from which red, green, and blue values could be extracted. Likewise, 14 out of 63 photos (22%) from the testing data set were unable to be processed due to failure to extract the QR code. The average red, green, and blue color values extracted from the training data set were used to train the predictive model.

### 3.2 Chlorine Concentration Measured by DPD-Based Test Strips Was Best Predicted by Using All Color Channels in a Polynomial Regression

**Figure 3:**
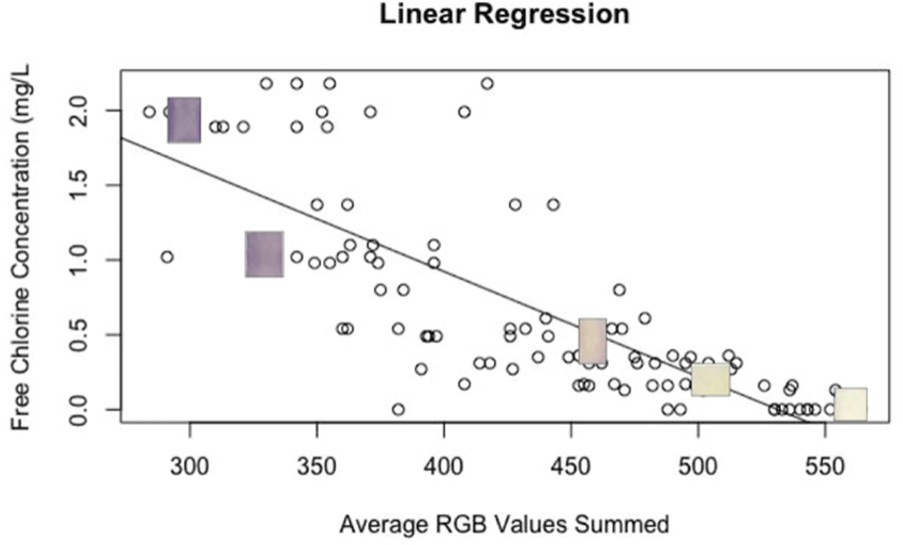
Relationship between RGB sum and chlorine concentration for free chlorine concentrations. Images of the free chlorine colorimetric pad have been inserted for concentrations of 2.0, 1.0, 0.5, 0.25, and 0.0 mg/L.

**Figure 4:**
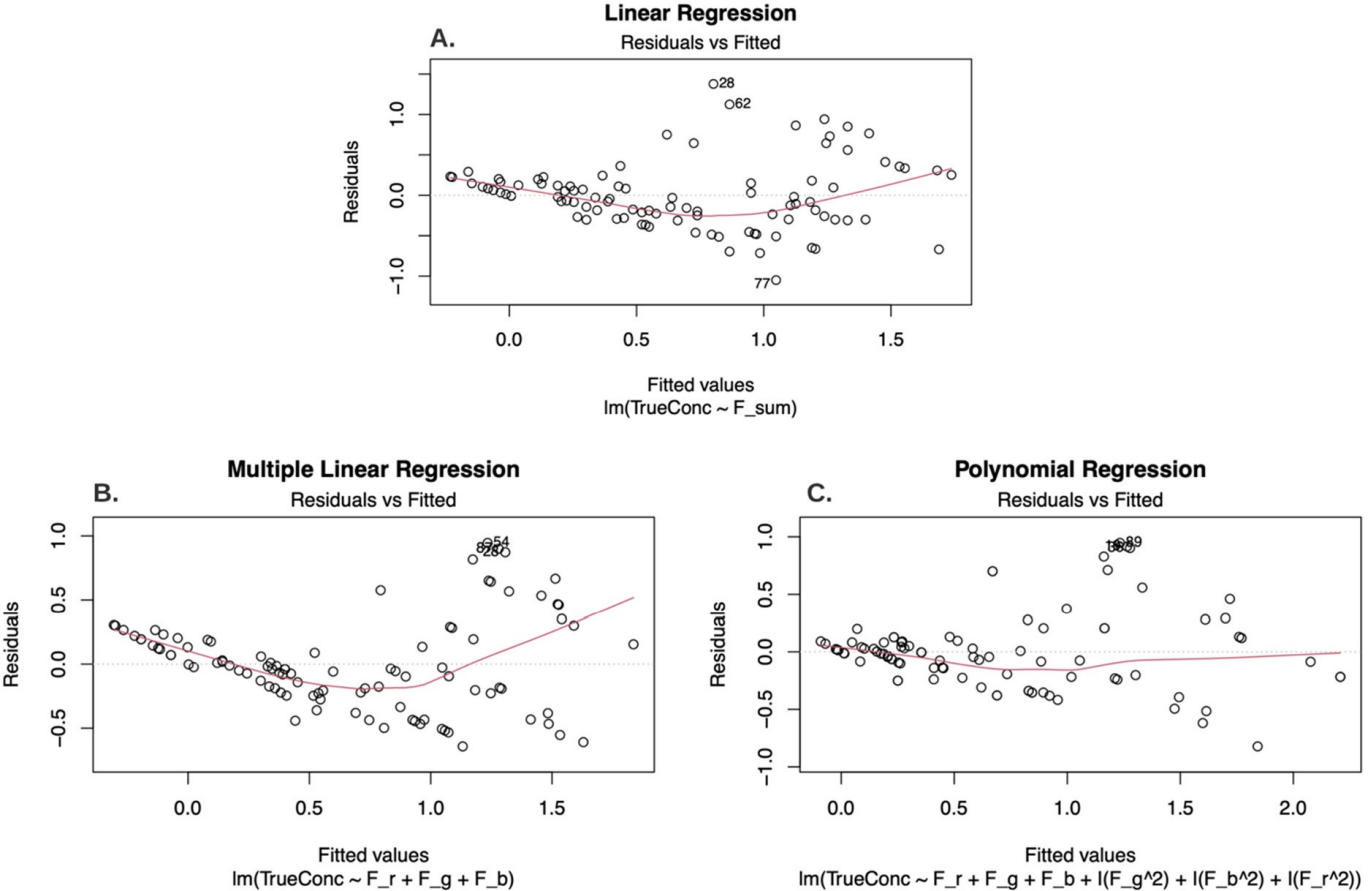
Residuals vs fitted plot for free chlorine training data using a linear regression (panel A), multiple linear regression (panel B), and polynomial regression (panel C). The TrueConc variable is the true free chlorine concentration. Any F_x variable corresponds to a free chlorine variable (e.g., F_r is the average red color value).

Multiple model structures were investigated to determine the best fit. An initial proof of concept evaluation using a linear regression model identified a negative relationship between the sum of the red, green, and blue color values in the colorimetric pad image and the known free chlorine concentrations in the training data set (Figure 3). This is to be expected as lighter colors (i.e., the colors produced by 0.5 mg/L or 0.2 mg/L) require more white (maximum value of 255); therefore, lower concentrations are associated with a larger RGB sum. The linear regression showed a relatively poor fit (*x* = −0.0070353, *b* = 3.7360509, *R*^2^_adj_ = 0.61), and the residuals vs fitted plot showed a general U-shaped pattern in the residuals, suggesting both over- and underestimation in the model (Figure 4, panel A).

A multiple linear regression was tested to observe if the model fit was improved when each channel was weighted individually. This regression model included only the red and green color values as independent variables because the blue channel was not statistically significant in the multiple linear regression (*p* = 0.138). The multiple linear regression model with red and green color values was a better fit (*x*_1_ = 0.015354, *x*_2_ = −0.026582, *b* = 1.878656, *R*_adj_^2^ = 0.71) than the linear regression model; however, the residuals versus fitted data plot was also U-shaped (Figure 4, panel B).

The U-shaped curve observed in the residuals indicated that a best fit model would likely be some form of polynomial regression. To identify the best fit model to predict free chlorine concentration, a backward stepwise regression was performed to minimize the AIC (Akaike information criterion) (Riley, 2009). This method identified a polynomial structure in which all three independent variables are included and squared (*x*_1_ = 6.2720e-02, *x*_2_ = −6.242 × 10^–2^, *x*_3_ = −1.3970 × 10^–1^, *x*_1_^2^ = 1.297 × 10^–4^, *x*_2_^2^ = 4.526 × 10^–4^, *x*_3_^2^ = −1.626 × 10^–4^, *b* = 1.086 × 10^1^, *R*^2^_adj_ = 0.75). It can be observed from the residuals versus fitted plot (Figure 4, panel C) that there remains a slight trend in the residuals; therefore, the residuals are not normally distributed (Shapiro–Wilk = 0.9753, *p* = 0.05). This is likely influenced by the fact that the distribution of data, while bell-shaped, is not Gaussian and deviates in the left and right tails. Further, it may be that the deviation in colors produced for different chlorine concentrations is not adequately sensitive to produce a model reliable enough to predict an absolute number at lower concentrations where it is known that the color variance is very difficult to discern by eye. This is consistent with the non-normality in the residuals, which indicates that the error in this model is not consistent across the full range of training data.

A common classification model, random forest (number of trees = 500; number of variables tried at each split = 2), was evaluated to determine if performance could be maintained or improved relative to the regression models investigated. Data were classed using binary (≤0.5 or >0.5 mg/L) and multiclass binning (≤0.2, 0.2–0.5, or >0.5 mg/L). The out of bag (OOB) estimate of the error rate for binary binning on training data was 7%. When applied to multiclass binning, the OOB estimate of the error rate on training data was 22%. These binnings were applied to the testing data with accuracies of 63% and 67%, respectively.

The relatively low accuracy of this random forest model when applied to testing data led us to consider an approach in which the polynomial regression model is coupled with a classification model. As observed by volunteers in a study investigating the accuracy, precision, usability, and cost of free chlorine residual testing methods, the discrimination of concentration by color is not easily determined using test strips by eye.^30^ When asked to determine the concentration of chlorine by eye, the mean percent error of volunteers was 54% for Hach brand test strips. Our own observations (248 data points total, including data gathered during preliminary methods development) yielded 50% accuracy when using the standard color grid provided by the test strips. Using colorimetric analysis to determine the explanatory variables in the model eliminates some of this error in color discrimination. We are ultimately interested in the ability of the toolchain to reliably determine if the concentration of free chlorine is less than 0.2 mg/L, between 0.2 and 0.5 mg/L, or above 0.5 mg/L, as these concentrations are widely used as minimums and a residual concentration below 0.2 mg/L must be reported to the EPA.^55^ Further, the goal of this work is not to understand how a unit change in an explanatory variable (red, green, or blue) changes the predicted outcome (free chlorine concentration), as this work does not propose a new colorimetric method. Therefore, while the polynomial regression model has non-normality in the residuals, we continued with this model coupled with a classification model to determine overall performance of the toolchain, as we are more interested in capturing bias in the classification outcome than in the predictors.

### 3.3 At Least 79% of the Free Chlorine Concentrations in the Training and Testing Data Sets Were Correctly Classified

The binary and multiclass binnings described above were applied to the polynomial regression results. For the binary binning, the positive class was defined as ≤0.5 mg/L; therefore, a false positive occurred when the binning model incorrectly predicts the negative class (>0.5 mg/L) as positive. Tables 2 and 3 show training and testing data set confusion matrices for the binary and multiclass binnings, respectively. Training and testing data set evaluative metrics for the binary and multiclass binnings can be found in SI Tables S11–S14.

**Table 2:** Training and Testing Data Set Confusion Matrices for Binary Binning^a^

	true class, mg/L			
	training		testing	
predicted class, mg/L	≤0.5	>0.5	≤0.5	>0.5
≤0.5	TP (46)	FP (9)	TP (23)	FP (1)
>0.5	FN (1)	TN (41)	FN (2)	TN (23)

a TP (true positive), FP (false positive), TN (true negative), FN (false negative). Numbers in parentheses are number of data points that fall into each bin.

**Table 3:** Training and Testing Data Set Confusion Matrices for Multi-class Binning^a^

	true class, mg/L					
	training			testing		
predicted class, mg/L)	≤0.2	0.2–0.5	>0.5	≤0.2	0.2–0.5	>0.5
≤0.2	TP (22)	2	0	TP (9)	1	0
0.2–0.5	8	TP (14)	1	2	TP (11)	1
>0.5	0	9	TP (41)	0	2	TP (23)

a TP (true positive), FP (false positive), TN (true negative), FN (false negative). Numbers in parentheses are number of data points that fall into each bin.

The accuracies for the binary binning was 90% and 94% for the training and testing data sets, respectively. The weighted-averaged F1-score for the multiclass binning was 79% for the training data set and 88% for the testing data set.

A closer look at the errors in the training data set reveals valuable information about both the regression and the classification model components. First, eight samples were predicted to have a negative free chlorine concentration by the regression model. Each of the eight samples were photos of test strips that had been used in a standard solution with no added chlorine; the known chlorine concentration was below detection according to the Hach DR900 meter, and there was no visual change in the colorimetric pads on any of the test strips. Therefore, these eight samples were considered true positives if classed in the lowest bin. All 8 samples were properly classified in both the binary and multiclass binnings. Second, the majority (9/10) of binary binning errors were false positives, or an underestimation, whereas the majority (17/20) of the multiclass binning errors were false negatives, or an overestimation. These overestimations were likely due to the fact that the ≤0.2 and 0.2–0.5 class recalls were not nearly as high as those of the >0.5 class, indicating that the model is less sensitive at concentrations below 0.5 mg/L, which is to be expected. Seven of the false negative samples were overestimated by a margin of ±0.1 mg/L or less: (0.49, 0.55), (0.17, 0.22), (0.17, 0.23), (0.16, 0.22), (0.16, 0.23), and (0.16, 0.25) as (true, predicted) in mg/L.

**Figure 5:**
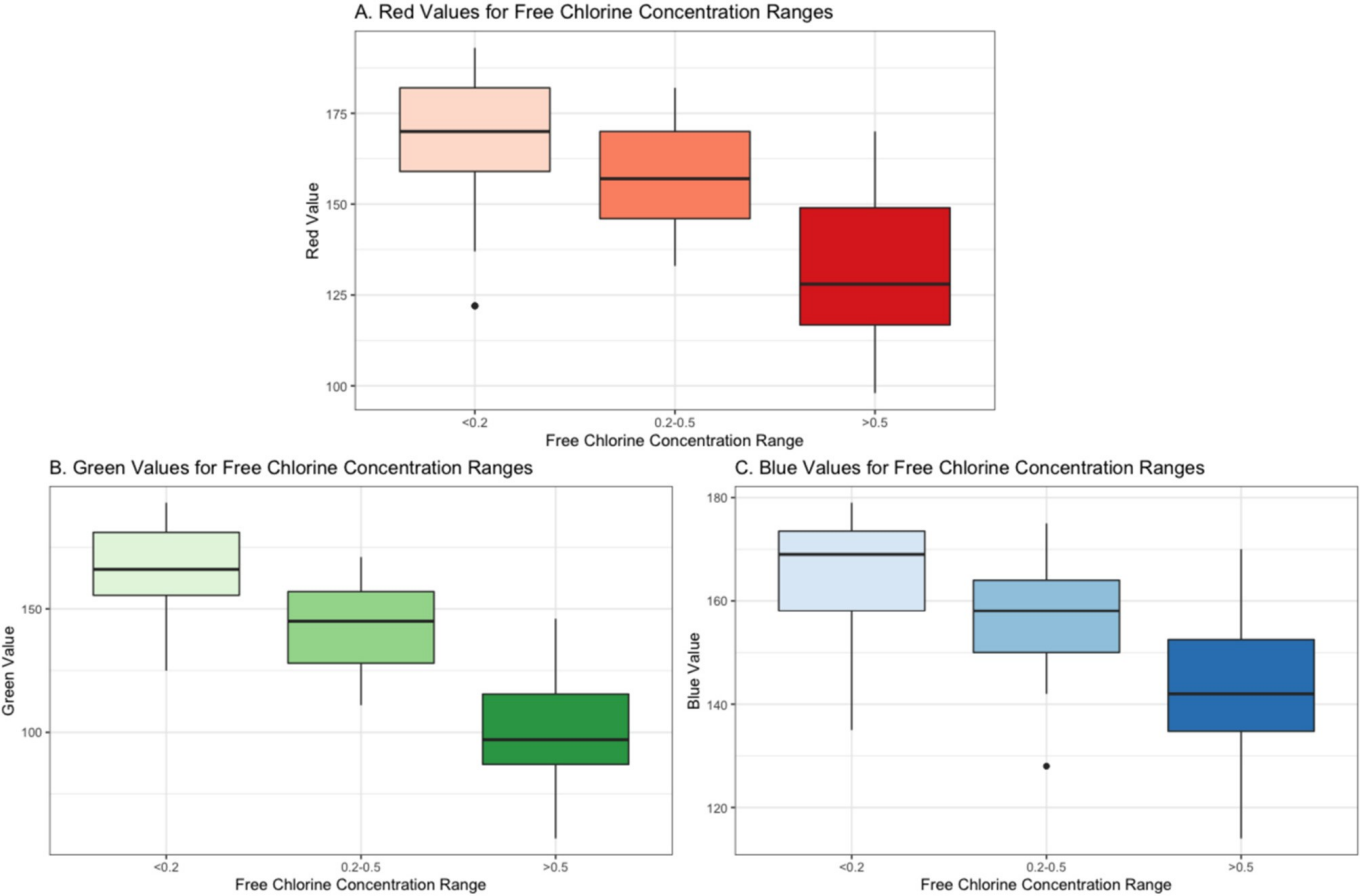
Boxplots indicating the range of red, green, and blue color values for free chlorine concentrations (panels A, B, and C, respectively). Maximum and minimum red color values for the three concentration ranges are 193–122, 182–133, and 170–98, in the order <0.2, 0.2–0.5, >0.5. Maximum and minimum green color values range from 193 to 125, 170–111, and 146–57, respectively. Maximum and minimum blue color values range from 179 to 135, 175–128, and 170–114, respectively.

There is a cost associated with inaccurately predicting concentrations at these levels. For example, a false negative in which a true concentration of ≤0.2 mg/L is predicted to be >0.2 mg/L underestimates the potential human health risk associated with insufficient disinfectant residual. As described above, the high rate of false negatives when using the multiclass binning indicates that model recall or sensitivity is lower at lower concentrations. As demonstrated in Figure 5, the range of observations for each color channel at concentrations less than or equal to 0.5 mg/L is smaller than those observed at higher concentrations (Figure 5, panels B and C) with the exception of the red channel (Figure 5, panel A). We hypothesize that adding more data to train the model at lower concentrations may improve model recall and decrease the number of errors.

### 3.4 Lighting and Phone Type Variation Can Be Successfully Accounted for Using White Balancing Techniques

**Figure 6:**
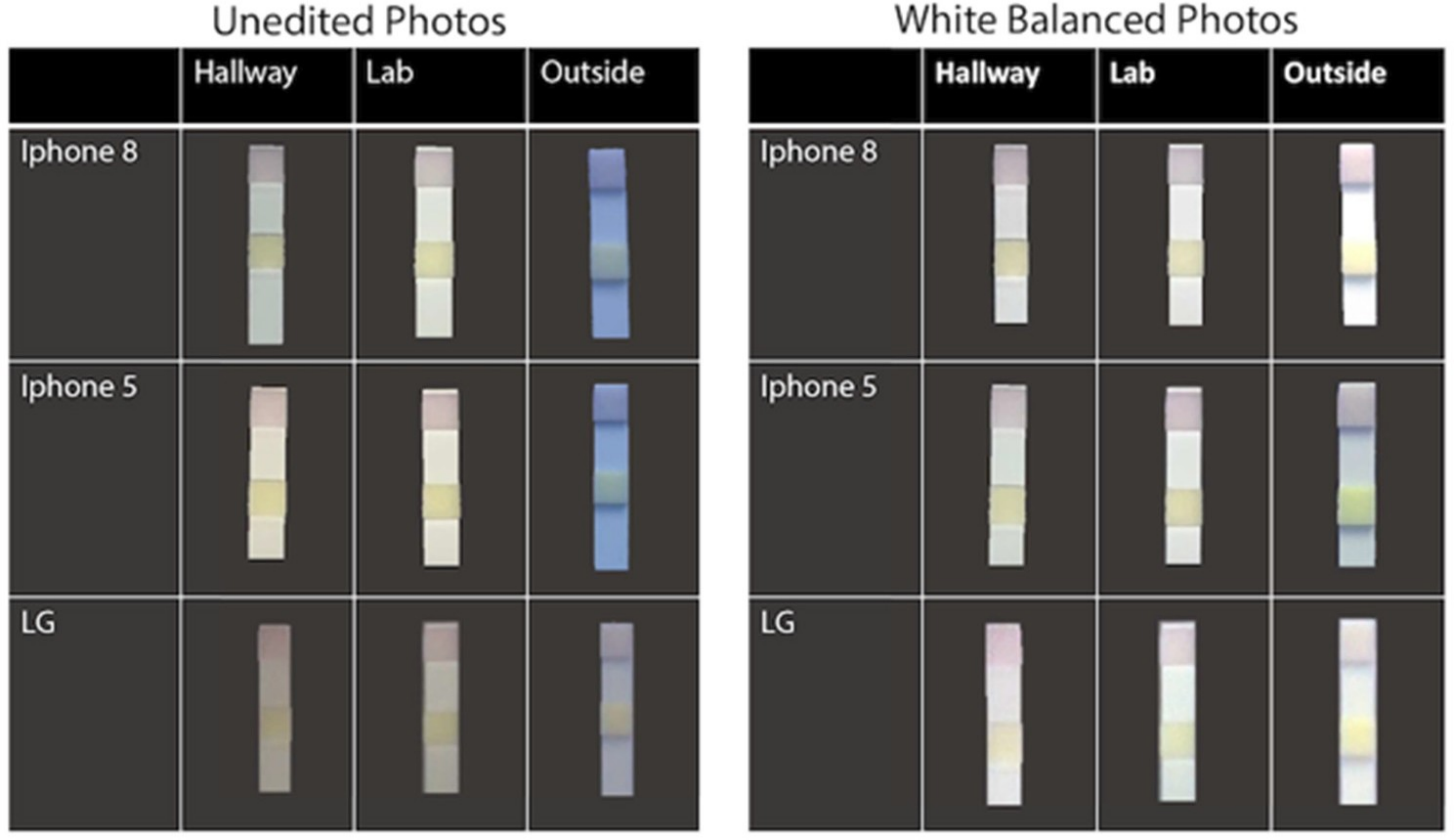
Test strip photos taken with three phone types (rows) and under three lighting conditions (columns) before and after normalizing via white balancing.

To determine whether lighting condition or phone type significantly influenced a data point’s likelihood of being classified correctly, a two-way z-test was performed. While LG enV2 photos were more likely to experience processing failure, photos taken in outdoor conditions were observed to have the most variance in overall hue (Figure 6). Z-test results showed that there was no statistically significant difference in percentage of correctly classified photos taken by any phone type (*p* = 0.46) or under different lighting conditions (*p* = 0.32) after white balancing was performed. Therefore, the white balancing methods used were successful in eliminating extreme red or blue coloring.

### 3.5 The Likelihood of a Misclassification Given Known Prior Probabilities Is Low

Conditional probability analysis can help to identify a classification error. To evaluate confidence in model results, we conducted a conditional probability analysis to examine the probability of a hypothetical present sample being classified as A given two prior samples in the same location are classified as B (assuming being classified as A is an error). We conducted this analysis to hypothesize performance in the field within one location (e.g., a neighborhood or a home with repeated prior samples). To calculate conditional probability, Bayes’ formula (eq 9) was used, which can be rewritten into the format of eq 10.
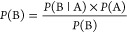
9
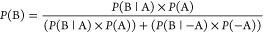
10where *P*(A|B) is the probability of A given B (or the posterior probability), *P*(A) is the probability of A (or the prior), *P*(B) is the probability of B (or the marginal probability), *P*(B|A) is the probability of B given A (or the likelihood), *P*(B|−A) = probability of B given not A, and *P*(−A) is the probability of not A.

To calculate prior probability, the combined training and testing results from the multiclass binning were used (Table 4). For example, if the true class is >0.5 mg/L, the probability of being classified as ≤0.5 mg/L is 0.14 based on prior observations (10/74 samples with a true concentration of >0.5 mg/L were misclassified as ≤0.5 mg/L in the combined data set). Further, if the true class is >0.5 mg/L, the probability of being classified as ≤0.2 mg/L is 0 based on prior observations (0/74 samples with a true concentration of >0.5 mg/L were misclassified as ≤0.2 mg/L in the combined data set).

**Table 4:** Combined Confusion Matrix for Multiclass Binning (Training and Testing Data)

	true class, mg/L		
predicted class, mg/L	≤0.2	0.2–0.5	>0.5
≤0.2	TP (31)	3	0
0.2–0.5	10	TP (25)	2
>0.5	0	11	TP (64)

Here, three cases were examined: the probability that the present sample is classified as ≤0.5 mg/L given two prior samples have been classified as >0.5 mg/L (Case 1), the probability that the present sample is classified as ≤0.2 mg/L given two prior samples are classified as >0.5 mg/L (Case 2), and the probability that the present sample is classified as >0.5 mg/L given two prior samples have been classified as ≤0.5 mg/L (Case 3).

The results for Cases 1, 2, and 3 are as follows: *P* < 0.02, *P* < 0.001, and *P* < 0.03, respectively. These results are encouraging for fieldwork. For example, Case 1 results suggest that the probability of a sample being classified as ≤0.5 mg/L given two prior samples have been classified as >0.5 mg/L is less than 2%. The low probability of this outcome suggests (a) a misclassification error of this type (an underestimate or false positive) is unlikely given prior probabilities and (b) if this outcome does occur in the field, it is worthwhile to check this measurement with a more sophisticated instrument, alert the drinking water utility that chlorine residual is low, or advise water users to take precautionary measures. Case 2 results demonstrate that the probability of the present sample being classified as ≤0.2 mg/L given two prior samples are classified as >0.5 mg/L is less than 1%. A misclassification of this type was not observed in any of the prior samples, and we have high confidence that it will not occur. Case 3 results indicate that the probability of a sample being classified as >0.5 mg/L given two prior samples were classified as ≤0.5 mg/L is less than 3%. The low probability of this outcome suggests, like Cases 1 and 2, that a misclassification error of this type (an overestimate or false negative) is unlikely. Therefore, should this outcome occur, the sample location may be of relevant interest to the utility, and if the chlorine concentration is very high, remediation activities may be necessary.

## 4 Scale-up and Proposed Future work

This work demonstrated proof-of-concept for an automated text-to-server toolchain that reliably captures free chlorine residual concentrations using chlorine residual test strips and a mid-to-high resolution mobile phone. We demonstrated the automation of the toolchain in which a photo of a test strip taken on a standard background can be white balanced and processed to isolate the colorimetric test strip pads. Further, the method used to predict and classify concentrations at ≤0.2 mg/L, 0.2–0.5 mg/L, or >0.5 mg/L was successful with a percent accuracy ranging from 79% up to 94%. Therefore, the method met the desired objective to improve reliability compared to volunteer estimations by eye and provide a means to store data for analysis.

This toolchain has limitations. First, 25% of the images in the complete data set were not processed due to failure to locate the QR code. It is hypothesized that most of these failures can be attributed to poor pixel resolution. Specifically, the legacy 12-year-old phone accounted for 25 of the 32 failures and had the lowest megapixel camera. We continue to iterate on the Python script to improve the robustness of the QR extraction process with lower resolution photos and reduce digital distortions, such that mobile phones with lower resolution cameras may still be used with the toolchain. Second, model sensitivity is relatively low at concentrations ≤0.5 mg/L compared to >0.5 mg/L, including between 0.2 and 0.5 mg/L. While this is likely due in part to the smaller variation in color produced at concentrations ≤0.5 mg/L, we also hypothesize that model sensitivity could be improved with more data points or alternative training methods, such as models that incorporate weighting. As acknowledged before, there is a cost to misclassifications (false negatives or false positives), and the consequences are not equivalent and should be carefully considered. For example, a false positive (underestimation of chlorine concentration) may cause undue stress to the water user and/or unnecessary remedial action (e.g., flushing or boosting). However, a false negative (overestimation of chlorine concentration) might obstruct the recognition of a potentially problematic condition (e.g., low disinfection residual), which may lead to an underestimation of public health risk and/or delay remedial action. In our work, it is of interest to produce a model that is both accurate and sensitive (high recall). When attempting to mitigate errors by reducing false positives and false negatives, the importance of protecting public health and the relatively minor consequences of a false positive result suggest that a highly sensitive model for low chlorine conditions is preferred, even if it occasionally results in false positives. Therefore, future work will seek to prioritize the resolution of false negatives by exploring methods to improve the toolchain performance at concentrations less than 0.5 mg/L.

Third, this toolchain was tested using lab-made free chlorine solutions free of possible interferences, such as elevated levels of manganese, and was not tested using combined chlorine solutions. DPD colorimetric chemistry is subject to interference from other oxidants if present in high concentrations. While methods are available to address interferences, they may not be feasible in the field and therefore have limited utility for a field-deployed monitoring program such as what we propose. To deploy this toolchain approach for user-based monitoring, it will be necessary to clarify the risk of chemical interferences in each community where it is deployed and to have a risk communication plan that can be enacted when false positives or negatives are found to occur so as to arm users with knowledge around uncertainty while not losing the value that more granular data and overall data transparency offers. Finally, we acknowledge that there are trade-offs, such as privacy concerns or information anxiety, that need to be thoughtfully considered with crowdsourced and publicly shared data sets.^56^ Increasingly, communities are organizing around the desire for effective community–academic partnerships that can enable data transparency while, at the same time, avoiding the kind of trade-offs noted.^57^

Future work includes piloting this toolchain within households across a community that is served by a chlorinated system; the data collected during the pilot will be further split into training and testing data to continue to train the model and improve model sensitivity. Once data are captured across a community, the data can be used to generate a temporal and geospatial map of free chlorine residual in premise plumbing over time, providing information about water quality to water users, utilities, and policymakers at a higher resolution than is currently available. This text-to-server toolchain sets the stage for future developments across the WDS that will provide opportunities for a more informed understanding of finished water quality at the point-of-use and better management of chlorine in drinking water mains. Further, this approach to sensing can be beneficial in areas where other sensing methods are not available or to supplement existing monitoring methods by extending coverage. We envision the implementation of this toolchain by utilities with test strips that retail for $0.70–1.30. The automated toolchain method can supplement their chlorine monitoring strategies at a lower cost than expanding monitoring locations or installing continuous online chlorine sensors that cost up to $2000 per sensor, not including sensor maintenance and operation.^2^ The resulting data can be used by the utility to select candidates for additional chlorine residual testing or areas in need of chlorine boosting if a household or community is consistently reporting low free chlorine concentrations.

## Supporting Information Available

The Supporting Information is available free of charge at and contains additional information about the various models; polynomial regression model sensitivity analysis; and both testing and training data (free and total chlorine) evaluative metrics. The Supporting Information is available free of charge at https://pubs.acs.org/doi/10.1021/acsestengg.2c00073.
