# Supplementary material for: An Automated Toolchain for Camera-Enabled Sensing of Drinking Water Chlorine Residual
Source: ACS ES T Eng. 2022 Jun 3;2(9):1697–708. doi: 10.1021/acsestengg.2c00073 (PMC9469768; doi:10.1021/acsestengg.2c00073)
Supplement: Supplementary file 1 — ee2c00073_si_001.pdf [file ee2c00073_si_001.pdf]

Supporting Information Cover Sheet

Authors: Alyssa Schubert, Leah Pifer, Jianzhong Cheng, Shawn P. McElmurry, Branko Kerkez,

Nancy G. Love

Manuscript Title: An automated toolchain for camera-enabled sensing of drinking water chlorine residual

Number of Pages: 9

Number of Figures: 3

Number of Tables: 14

Supporting Information for: An automated toolchain for camera-enabled sensing of drinking water chlorine residual.

S1. Comparison of model performance by model type.

A summary of performance (percent accuracy) for all models tested in this work is presented in Table S1.

Table S1. Comparison of model performance by model type. Percent accuracy is rounded to the nearest percent.

	**Percent Accuracy**			
**Model Type**	**Free Chlorine Training Data**<br><br>Binary Binning	**Free Chlorine Testing Data**<br><br>Binary Binning	**Free Chlorine Training Data**<br><br>Multi-Class Binning	**Free Chlorine Testing Data**<br><br>Multi-Class Binning
**Linear Regression**	77	86	64	67
**Multiple Linear Regression**	90	96	78	90
**Polynomial Regression**	90	94	79	88
**Random Forest**	93	63	78	67

S2. Added variable (partial regression) plots for the multiple linear regression and polynomial regression models.

Added variable plots can be useful to visualize regressions with multiple predictors. Figure S1shows the added variables plots for the multiple linear regression. **Error! Reference source not found.** shows the added variable plots for the polynomial regression model. Each plot demonstrates the relationship between each predictor variable and the outcome variable, all other

predictors (“others”) held constant. For example, the y-axis may be interpreted as “TrueConc given all other predictors are accounted for.” In Figure S1, the upper left graph shows the relationship between the free chlorine average red color values and the true concentration, all other predictors held constant. There are no obvious outliers and the slope of the plot is positive, meaning that an increase in the red value is associated with an increase in the true concentration, all other predictors held constant. All other added variable plots can be interpreted in the same manner.

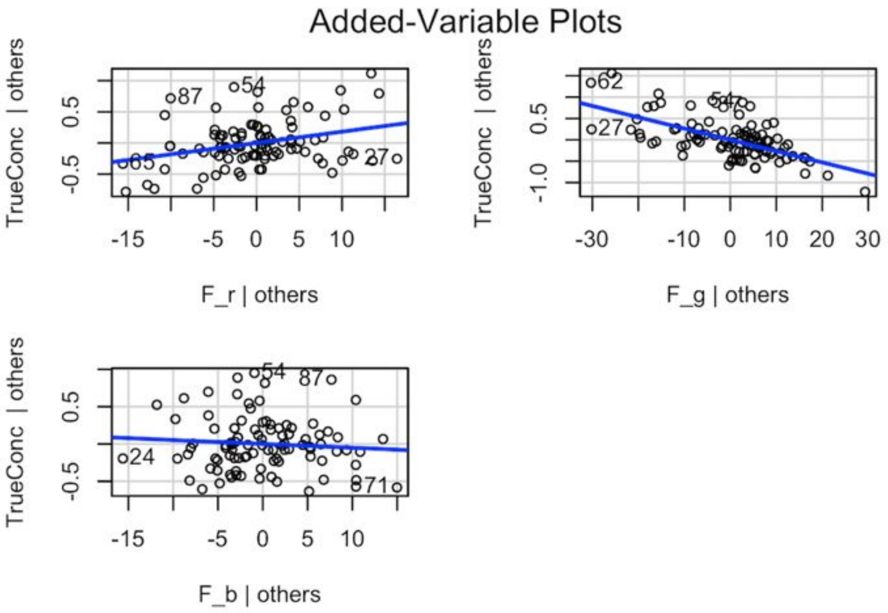

Figure S1. Added variable plots for the multiple linear regression model. F_r is the average red color value for a free chlorine colorimetric pad. Likewise, F_g and F_b are the average green and blue color values, respectively.

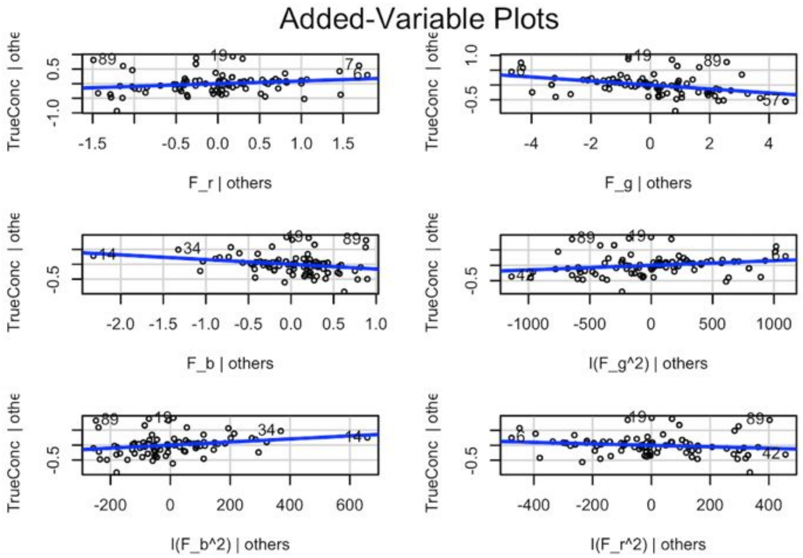

Figure S2. Added variable plots for the polynomial regression model. F_r is the average red color value for a free chlorine colorimetric pad. Likewise, F_g and F_b are the average green and blue color values, respectively. I(predictor^2) is the square of each predictor.

S3. Additional model data.

Figure S3 shows the distribution of red, green, and blue color values across free chlorine concentrations.

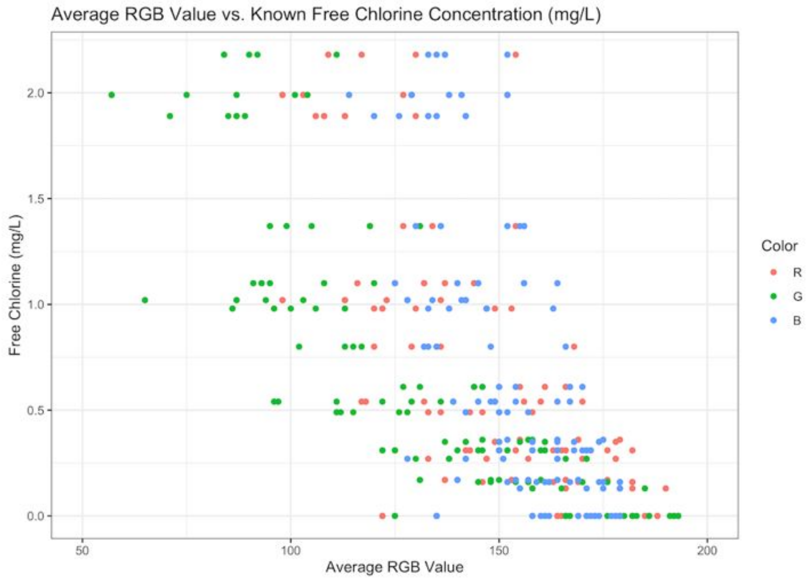

Figure S3. Graph of average red, green, and blue color values vs. free chlorine concentration (mg/L).

Table S2 describes in detail the number of samples per lighting and phone type condition.

Table S2. Distribution of photos taken per condition tested.

	**Phone Type**			
**Lighting Condition**	**LG EnV2**	**iPhone 5**	**iPhone 8**	**Total**
**Laboratory**	21	21	23	65
**Hallway**	20	21	22	63
**Outside**	20	21	23	64
**Total**	61	62	68	192

Model results for free chlorine were cross-validated by resampling the complete dataset ten times, while preserving relative proportionality, to generate 10 new training datasets. Percent accuracy for both the binary and multi-class binnings were calculated for each of the training datasets (Table S3).

Table S3. Percent accuracy of classification of free chlorine concentrations for both binning structures, calculated using randomly generated training datasets 1-10.

Dataset	Percent Accuracy (Binary)	Percent Accuracy (Multi-class)
Training Dataset 1	89.7	75.3
Training Dataset 2	93.8	81.4
Training Dataset 3	90.7	77.3
Training Dataset 4	91.8	81.4
Training Dataset 5	89.7	77.3
Training Dataset 6	93.8	84.5
Training Dataset 7	90.7	78.4

Training Dataset 8	92.8	83.5
Training Dataset 9	89.7	79.4
Training Dataset 10	90.7	77.3

The percent accuracy for the binary binning ranges from (89.7, 93.8) with a mean of 91.3, median 90.7, and standard deviation of 1.62 percent. The percent accuracy using the multi-class binning ranges from (75.3, 84.5) with a mean of 79.6, median 78.9, and standard deviation of 3.00 percent. The percent accuracies associated with the dataset used to train the model (89.7 and 79.4 for the binary and multi-class binnings, respectively) both fall within one standard deviation of the respective means.

Table S4 was used to calculate the conditional probabilities for Cases 1-3. Reading across the table gives the number of photos in a true concentration bin vs. the number of those photos classified in each bin (e.g., 32/36 photos with a true concentration of <0.2 mg/L were classified as <0.2 mg/L; 4/36 photos with a true concentration of <0.2 mg/L were classified as 0.2-0.5 mg/L). Reading down the table gives the number of photos classified in a bin vs. the number of those photos with a true concentration in each bin (e.g., 32/41 photos classified as <0.2 mg/L had a true concentration of <0.2 mg/L).

Table S4. Combined results (testing and training data) using the multi-class binning structure.

	**True Class**			
**Predicted Class**	**< 0.2 mg/L**	**0.2-0.5 mg/L**	**>0.5 mg/L**	**Total**
**< 0.2 mg/L**	32	9	0	41
**0.2-0.5 mg/L**	4	25	10	39
**>0.5 mg/L**	0	3	64	67
**Total**	36	37	74	147

S4. Total chlorine training and testing data results.

The total chlorine data were excluded from the main analysis because the data were collected using only free chlorine standard solutions; therefore, this work has not directly investigated the reliability of the toolchain to predict and classify total chlorine concentrations with chloramine and other combined chlorine solutions. However, an analysis was completed to understand how well the model performed to predict and classify total chlorine concentrations in free chlorine solution. Tables S5 and S6 are the resulting confusion matrices for both training and testing data. Tables S7 through S10 describe the evaluative metrics used to analyze model performance with total chlorine data.

Table S5. Training and testing dataset confusion matrices for binary binning.

	**True Class (mg/L)**			
	**Training**		**Testing**	
**Predicted Class (mg/L)**	**≤ 0.5**	**> 0.5**	**≤ 0.5**	**> 0.5**
≤ 0.5	TP (50)	FP (5)	TP (22)	FP (3)
> 0.5	FN (4)	TN (38)	FN (2)	TN (22)

Table S6. Training and testing dataset confusion matrices for multi-class binning.

	**True Class (mg/L)**					
	**Training**			**Testing**		
**Predicted Class (mg/L)**	≤ 0.2	0.2-0.5	> 0.5	≤ 0.2	0.2-0.5	> 0.5
≤ 0.2	TP (14)	7	0	TP (2)	5	0
0.2-0.5	16	TP (13)	4	8	TP (7)	2

> 0.5	0	5	TP (38)	1	2	TP (22)

Table S7. Training and testing dataset evaluative metrics for binary binning.

**Dataset**	**Accuracy**	**Precision**	**Recall**	**F1-score**
**Training**	0.91	0.91	0.92	0.92
**Testing**	0.90	0.88	0.92	0.90

Table S8. Training dataset evaluative metrics for each class in the multi-class binning.

Class	Accuracy	Precision	Recall	F1-score
≤ 0.2	0.79	0.67	0.47	0.55
0.2-0.5	0.65	0.39	0.52	0.45
> 0.5	0.91	0.88	0.90	0.89

Table S9. Testing dataset evaluative metrics for each class in the multi-class binning.

Class	Accuracy	Precision	Recall	F1-score
≤ 0.2	0.71	0.29	0.18	0.22
0.2-0.5	0.65	0.41	0.5	0.38
> 0.5	0.90	0.88	0.92	0.90

Table S10. Training and testing dataset overall evaluative metrics for multi-class binning.

**Dataset**	**Total Accuracy**	**Total Precision**	**Total Recall**	**Weighted-Averaged F1-score**
**Training**	0.67	0.67	0.67	0.67
**Testing**	0.63	0.63	0.63	0.60

Training and testing data accuracy for the binary binning was 91% (Ac=88/97) and 90% (Ac=44/49), respectively, which does not deviate significantly from the accuracy calculated for the free chlorine data and further supports significant improvement in the ability to distinguish concentrations above and below 0.5 mg/L compared to estimates by eye. The weighted-averaged F1-score for the multi-class binning was 67% for the training data and 60% for the testing data. These results are relatively poor compared to the free chlorine multi-class results, although they follow the trend set by free chlorine data in that they underperform the total chlorine binary binning results. In both training and testing datasets, precision and recall were greatest for the > 0.5 class, suggesting that this model performs well at classifying total chlorine concentrations greater than 0.5 mg/L. At lower concentrations, model sensitivity, or recall, is quite low, in particular when classifying concentrations ≤ 0.2 in the testing dataset, in which 9 out of the 11 samples in that class were overestimated. We hypothesize that this diminished multi-class performance compared to the free chlorine results is likely because the color produced on the total chlorine colorimetric pad has less variation than the free chlorine pad. In sampling, it was observed that the color produced by the total chlorine pad was darker than the color produced for the same concentration by the free chlorine pad. This is because the color chemistry is different; the total chlorine pad is imbued with iodine in addition to DPD. The model results also suggest that the model should be trained using combined chlorine solutions to understand how model performance might change. Continued analysis with combined chlorine solutions is needed to draw further conclusions.

S5. Free chorine training and testing confusion evaluative metrics for binary and multi-class binning.

The following tables summarize the evaluative metrics for free chlorine training and testing data.

Table S11. Training and testing dataset overall evaluative metrics for binary binning.

Dataset	Accuracy	Precision	Recall	F1-score
**Training**	0.90	0.84	0.98	0.90
**Testing**	0.94	0.96	0.92	0.94

Table S12. Training dataset evaluative metrics for each bin in the multi-class binning.

Class	Accuracy	Precision	Recall	F1-score
≤ 0.2	0.90	0.92	0.73	0.82
0.2-0.5	0.79	0.61	0.56	0.58
> 0.5	0.90	0.82	0.98	0.89

Table S13. Testing dataset evaluative metrics for each bin in the multi-class binning.

Class	Accuracy	Precision	Recall	F1-score
≤ 0.2	0.94	0.90	0.82	0.86
0.2-0.5	0.88	0.79	0.79	0.79
> 0.5	0.94	0.92	0.96	0.94

Table S14. Training and testing dataset overall evaluative metrics for multi-class binning.

Dataset	Accuracy	Precision	Recall	Weighted-Averaged F1-score
**Training**	0.79	0.79	0.79	0.79
**Testing**	0.88	0.88	0.88	0.88
